# Extraction of Teeth and Facial Paralysis

**Published:** 1897-07

**Authors:** 


### Extraction of Teeth and Facial Paralysis.

In a paper on "Extraction of Teeth and Facial Paralysis," Dr. Frankl Hochwart gives an account of six cases which he has observed. In the first case the patient had had an attack of facial paralysis seven years before, and the second attack, which affected the same side, came on the day after the extraction of teeth and without any other complication, and in these cases the paralysis was on the same side as the extraction. So it was in the sixth case, while in the fifth it was on the opposite side. Dr. Hochwart does not regard the actual extraction as the direct cause of the paralysis, but rather the condition of inflammation which renders extraction necessary or, at least, desirable; and he points out the fact that injury to a tooth may cause paralysis in anyone with a predisposition, as was the case in a young woman who suffered from a third attack of facial paralysis after the accidental breaking of an incisor. He also thinks that inflammation about the teeth may cause paralysis even if extraction has not been done.—*Lancet*

